# Study on anaerobic phosphorus release from pig manure and phosphorus recovery by vivianite method

**DOI:** 10.1038/s41598-023-43216-5

**Published:** 2023-09-26

**Authors:** Tengshu Chen, Xingfu Song, Mengyao Xing

**Affiliations:** 1 College of Resource and Environmental Science, Key Laboratory of Rural Environmental Remediation and Waste Recycling, Quanzhou Normal University, Dong Hai Street, Feng Ze District, Quanzhou City, 362000 Fujian Province China; 2 Department of Advanced Manufacturing, FuZhou University, No. 1, ShuiCheng South Road, Jinjiang, 362200 Fujian China; 3 Department of Architecture ArtsGuangxi Art College, No. 8 Luowen Avenue, Xixiangtang District, Nanning, 530000 Guangxi China

**Keywords:** Environmental sciences, Chemistry

## Abstract

In this study, pig manure rich in phosphorus was used as the recovery object, In order to realize the maximum recovery of phosphorus resources in pig manure, this study established a phosphorus recovery route combining the electrochemical method with the Vivianite method using sacrificial iron anode. And in order to obtain phosphorus rich supernatant, pig manure was treated with different pH values, and the changes in phosphorus components and metal content in the liquid phase were mainly investigated; Graded phosphorus components and microbial communities in the solid phase; Finally, the effect of electrolytic recovery of phosphorus from fermentation supernatant was studied. The results showed that the highest total phosphorus (TP) content in the liquid phase follows a trend of acidity > control > alkalinity; The analysis of the results of solid-phase phosphorus fractionation extraction shows that acidic conditions are more conducive to the release of Non-apatite inorganic phosphorus (NAIP) and Apatite inorganic phosphorus (AP); The microbial community promotes the release of phosphorus by participating in the decomposition of fermentation substrates; The analysis of the change of metal content in the liquid phase before and after electrolysis showed that the two chamber electrolytic cell can not remove other metal components while recovering the vivianite; More than 90% of the phosphorus in the supernatant after fermentation was recovered by electrolysis. The characterization results showed that 84.66% of the precipitate was Vivianite.

## Introduction

With the large-scale and intensive development of China's pig breeding industry, the wastewater produced in the process of breeding will pollute the water. The problem of pollution has become increasingly serious and has become the main source of pollution in rural areas of China. The Chemical Oxygen Demand (COD) of the wastewater is 5000–20,000 mg/L, the ammonia nitrogen (NH_3_–N) is 600–1600 mg/L, the P concentration is 100–250 mg/L, and it carries a large number of pathogenic bacteria, giving off a very strong odor. Generally, livestock manure is regarded as a nutrient resource pool because it contains a large amount of organic matter and is the source of nitrogen (N), P and kalium (K)^1^. However, the total P loss of China increased from 0.2 million tons in 1950 to 3.1 million tons in 2010^2^, most of which was due to the direct release of animal manure from livestock farms into water^3^, on the one hand, excessive P discharged into the water will cause the waste of resources, and on the other hand, it will also cause eutrophication of water. In addition, the Ministry of Electricity and Water Resources has studied the reserves and consumption of P, and they predict that the global P ore resources will be exhausted in the next 50–100 years, resulting in a surge in the price of P ore market^4^.

Previous studies have shown that phosphate can be removed from wastewater by chemical precipitation, biological polyphosphate accumulation or the combination of the two methods^5^. Chemical P removal includes adding Ca, Fe or Al to combine phosphate to form precipitation^6^. Pig manure also contains a large amount of phosphate. Most of the P will be released into the supernatant after treatment. Some researchers used high-pressure homogenizer for pretreatment, and the concentration of TN and TP in the sludge supernatant increased by 10 times and 3 times respectively. And others use ultrasonic degradation to treat activated sludge to promote the dissolution of more than 60% of TP^7^.During anaerobic digestion, different treatment methods can achieve phosphorus release efficiencies of up to 64% and 63%, respectively^8^. Previous studies have shown that pH is one of the main factors affecting P release during anaerobic digestion, because it affects the adsorption–desorption and precipitation–dissolution processes of solid P^9^. Most of the P released during the fermentation process is in the form of phosphate (PO_4_^3−^). Furthermore, with the progress of hydrolysis acidification, the P concentration also increased rapidly, and PO_4_^3−^–P increased from 50L to 280 mg/L 20 days later^10^. The excrement contains a large amount of P, and most of the treated P is free state, which is convenient for subsequent recycling. The phosphorus content in pig manure is closely related to the cleaning method. The most common defecation processes in intensive pig farms in China include dry defecation, water flushing, and water soaking. Dry defecation has high value as a manure fertilizer, making it easy to compost at high temperatures. However, most of the phosphorus is present in the manure and cannot be directly utilized; Water flushing has advantages such as low labor intensity and high efficiency, but the amount of wastewater is large, the concentration of pollutants is high, and the phosphorus content in wastewater is low; Soaked feces have low labor intensity and high efficiency. Long term soaking of feces can create conditions similar to anaerobic fermentation, promote phosphorus release, and have a high phosphorus content in the supernatant. Due to the significant impact of different defecation methods on phosphorus release in pig manure, this study adopted a pre-treatment method of adding water to dry manure and soaking manure.

The emerging vivianite recovery method can only be formed in an environment rich in P and iron. The optimum formation pH is 6–8^11^. Vivianite has high economic value, the price of vivianite is about 10,000 €/t. And it is the basic raw material for the production of lithium iron phosphate (LiFePO_4_), Lithium iron phosphate is increasingly used as a precursor in the manufacture of lithium-ion secondary batteries^12,13^. The use of vivianite as a pigment can be traced back to the thirteenth and fourteenth centuries^14,15^. And large high-purity vivianite has high collection value^16^. At the same time, vivianite also plays an important role in Heavy metal fixation (HMs), carbon tetrachloride (CT) dechlorination, P retention and eutrophication mitigation^17^.Therefore, the recovery of P by the vivianite has great development potential.

The previous experiment successfully set up a two-chamber electrolytic cell and successfully recovered more than 97% of P in the form of vivianite^18^. This study has established a phosphorus recovery route combining the electrochemical method using sacrificial iron anode and the Vivianite method. Compared with other Vivianite synthesis methods, it can achieve rapid phosphorus removal, wide application range, simple operation, easy collection of products and other advantages. This experiment is the follow-up work of the previous experiment. The main research content is to take pig manure as the object, conduct anaerobic fermentation through pH adjustment to obtain P-rich supernatant, and then use the two-chamber electrolytic tank to treat the supernatant, demonstrating the feasibility of the two-chamber electrolytic tank to recover vivianite for actual sewage treatment. .The experiment was made in triplicate and an average value was reported.

## Materials and methods

### Pig manure source and treatment method

**Figure 1:**
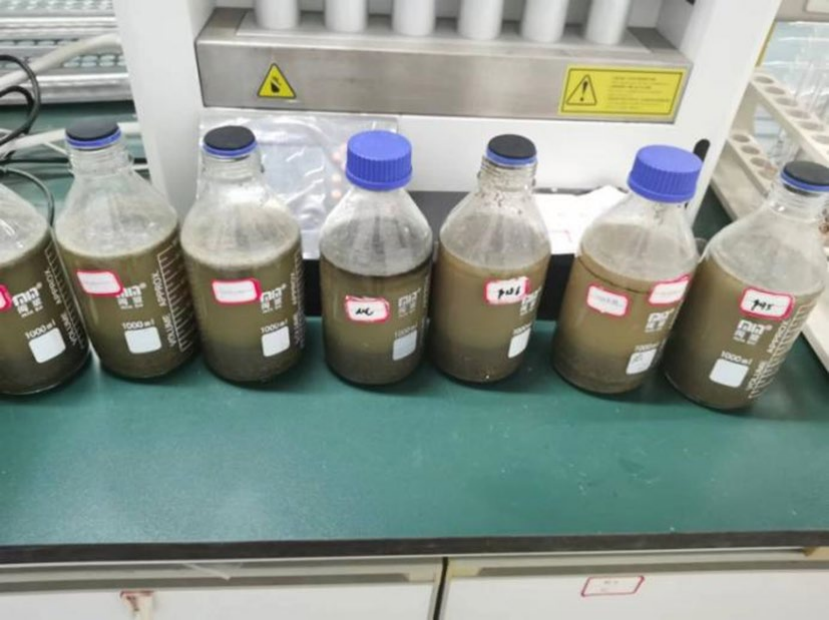
Schematic diagram of anaerobic fermentation of pig manure.

The pig manure used in the experiment was from a small pig farm in Quanzhou. Taking dry pig manure from the pig farm and prepare seven 1000 mL blue cap bottles for standby. As shown in Fig. 1, take the same amount of pig manure and put it into a blue cap bottle, add the same amount of ultra-pure water to 1000 mL to prepare 1000 mL of pig manure water solution. Do not add other ingredients to the solution to eliminate the interference of other factors, and then put it in shaking incubator for 30 min to shake it up. Then use 3 M NaOH and HCl to adjust the pH to 5.0, 6.0, 7.0, 8.0, 9.0, 10.0, and the unadjusted group as the none control (NC), the pH of the NC is 7.6 ± 0.23. Cover the rubber plug and place it in the oscillation box. The temperature is set at 35 ± 1℃ and the rotational speed is 150 rpm for 7 days. During the Anaerobic Fermentation (AF) process, the inoculum was not added to the bottle, and the fermentation supernatant was taken every 24 h to measure the P content in the solution. After fermentation, the mixed aqueous solution was centrifuged at 4000r/min for 15 min, and the supernatant was collected and placed in a refrigerator at 4℃ for subsequent electrolysis experiments. The sediment is collected and placed in a freeze dryer for freeze drying. Then grind through a 100-mesh sieve, extract the solid P components in the sediment, and determine the content of different P components.

### Preparation for electrolytic experiment

The experimental instrument is a two chamber electrolytic cell made in the laboratory. The supernatant after fermentation is taken as the anode solution, and 4 g/L sodium chloride is added to ensure the ionization strength. The cathode liquid is prepared into sodium chloride aqueous solution with 4 g/L sodium chloride, and the anode and cathode chambers are separated by proton exchange membrane (Nafion 117). Effective utilization area of proton exchange membrane is 0.69π cm^2^.The anode material is pure iron plate (0.5 mm × 50 mm × 100 mm), and the cathode material is titanium plate of the same specification.

### Calculation and measurement method

Electrolyte the supernatant and take samples every one hour, filter with 0.45 μm filter membrane, determine the residual soluble P(PO_4_^3−^–P ) in the solution by ammonium molybdate spectrophotometry, and the P recovery rate(η_P_) in the solution calculated by formula (1):

1 $$\eta_{P} = \frac{{C_{0} - C_{t} }}{{C_{0} }} \times 100\%$$

Where, C_0_ (mg/L) is the initial P concentration, C_t_ (mg/L) is the P concentration at the time of sampling.

The Standards,Measurements and Testing (SMT) method is used for the analysis and extraction of solid P^19^. After the reaction, the sample is collected from the bottom of the reactor, put it in a centrifuge and centrifuge at 4000r/min for 10 min, the supernatant is separated and stored for later use, collected for precipitation, and then freeze-dried in a vacuum freeze dryer for 48 h. The powder precipitate obtained is sent to a special testing agency for testing. The crystal form of precipitation was characterized by XRD, and the scanning speed was 4.0°/min, the range of 2θ is 10°–60°, and the obtained atlas is analyzed with MDI Jade 6 software. The morphology of the product was observed by SEM, and its elemental composition was analyzed by EDS. Fourier transform infrared spectrometer (FTIR) is used to analyze chemical bonds in products. The purity of vivianite is calculated by formula (2):

2 $${\text{K}} = \left( {1 - \left| {\frac{1 - \text{X}}{1.5}} \right|} \right) \times 100\%$$

where, K is the purity of vivianite calculated according to EDS analysis, X is the atomic ratio of Fe/P in the surface precipitate, and the precipitation result shows that it is 1.27.

### Ethics approval and consent to participate

This study did not use any kind of human participants or human data, which requires any kind of approval. All authors agreed to contribute to this study.

## Results and discussion

### Changes of P components in liquid phase

**Figure 2:**
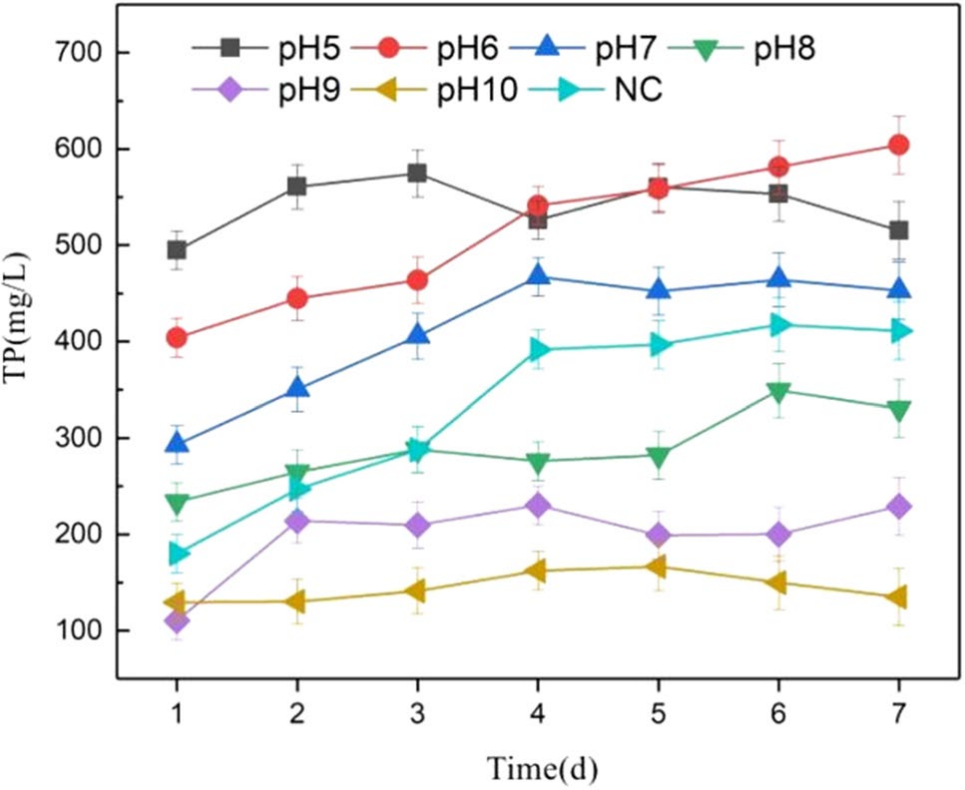
Changes of phosphorus components in liquid phase.

The anaerobic fermentation of pig manure is carried out by adjusting different pH, and the fermentation supernatant is analyzed and demonstrated to evaluate the effect of different pH on the anaerobic fermentation of pig manure and the P release mechanism. As shown in the Fig. 2: the experimental results show that the effect of promoting P release under different pH conditions is divided into acidic zone and alkaline zone with NC group as the boundary. In the acid zone (pH is 5–6), on the first day of fermentation, the P in the liquid phase rose to 494.7 mg/L and 403.9 mg/L respectively, and as time went on, the P release under pH = 5 conditions reached a high level on the third day, and there was no sharp increase in the subsequent period of time, and it remained stable within the error range. On the 7th day after fermentation, the final P content was 515.3 mg/L, 1.25 times higher than 411.1 mg/L in the NC group. Under the condition of pH = 6, the P content continued to rise in the following period, and the final content remained at 604.3 mg/L, 1.47 times higher than that of the NC group 411.1 mg/L.

At pH = 8, pH = 9 and pH = 10, the P content increased to 233.8 mg/L, 110.3 mg/L and 129.5 mg/L respectively on the first day of fermentation. When the fermentation reached the the fourth day, the P content rose to a larger value in the pH range, and the P content did not increase rapidly in the subsequent period, it remained within the relatively stable error range, and the final P release was lower than the control group. This phenomenon shows that in the anaerobic fermentation of pig manure, acid treatment is beneficial to the release of P, while alkaline treatment is not conducive to the release of P.

In summary, speaking, the P release effect generally follows the trend of acid > neutral > control > alkaline. The final P release under acidic conditions was greater than that of the control group, while the final P release under alkaline conditions was lower than that of the control group. It also follows the trend that the higher the pH, the less the P release. Therefore, it can be concluded that promotion effect of anaerobic fermentation of pig manure under acidic conditions is better than that under alkaline conditions.

### Change of solid P component

**Figure 3:**
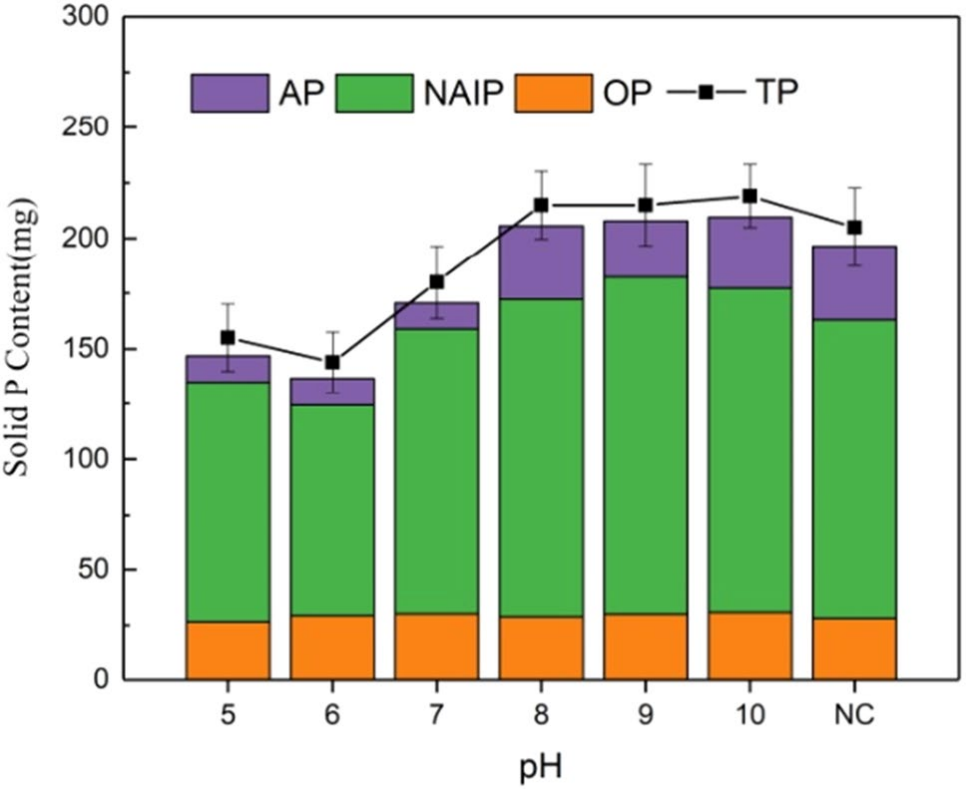
Distribution diagram of solid phosphorus components.

After the end of anaerobic fermentation by adjusting pH, the P content in the solid phase of pig manure decreased to varying degrees. This also confirmed that acid–base regulation is also conducive to the release of P in sludge. As shown in the Fig. 3, Organic phosphorus (OP) content after fermentation still occupies a small part. After pH adjustment, the residual P content in pig manure is 30 mg/L, regardless of acid and alkali conditions, which proves that acid and alkali adjustment has similar effect on promoting the release of OP, while OP under acidic conditions is slightly lower than OP under alkaline conditions, because acidity is more conducive to the disintegration of small and medium-sized particles in pig manure.

NAIP is still the component with the highest content in the solid P component. On the whole, the content of NAIP under acidic conditions is lower than that under alkaline conditions, and the content of NAIP under alkaline conditions is also higher than 134.9 mg/L of the NC group, indicating that the promotion of NAIP release effect under alkaline conditions is weak. The P release of NAIP under acidic conditions is mainly due to the leaching of metal ions, and the release of NAIP under alkaline conditions depends on the competition between OH^−^ and PO_4_^3−^ adsorption sites. The residual content of AP under acidic conditions is far less than that under alkaline conditions. Under alkaline conditions, the content of AP remains stable, indicating that alkaline conditions are not conducive to the release of AP, and even lead to the formation of AP. AP is usually used as calcium-bound P of non-active P. Under acidic conditions, Ca^2+^ is leached by acid to release calcium-bound P. However, under alkaline conditions, the release amount is less or even no, indicating that alkaline conditions are not suitable for AP release.

### Microbial population and abundance

**Figure 4:**
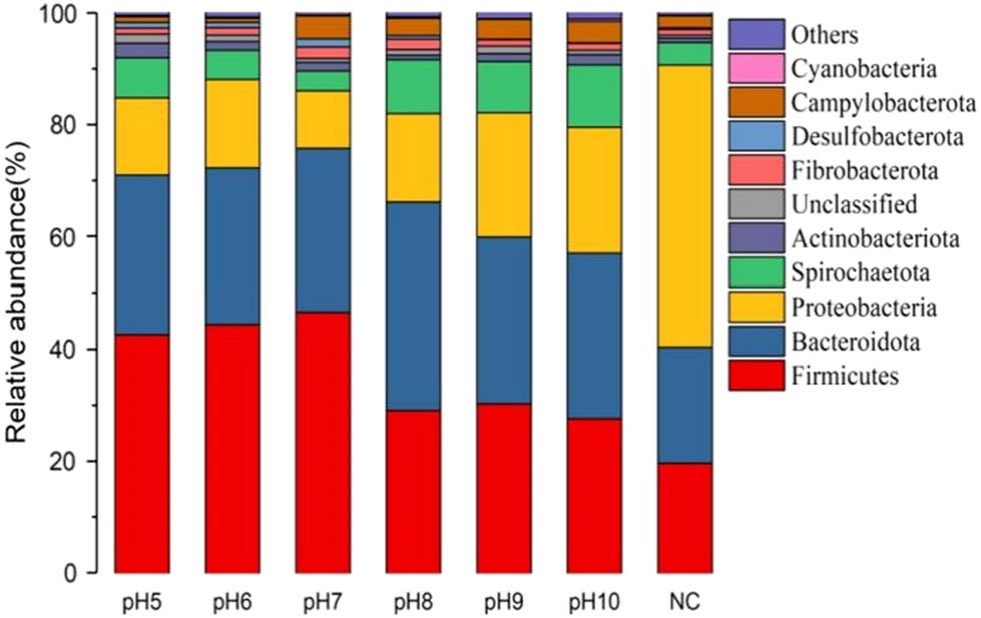
Relative abundance distribution of microbial community at phylum level under different pH conditions.

In order to further analyze the impact of different pH pretreatment conditions on functional microorganisms, the distribution of microbial communities was analyzed at the phylum level (Fig. 4). *Firmicutes, Proteobacteria, Bacteroidetes, Spirochaetes* are the main microorganisms in the anaerobic digestion system^20–22^. In the sludge of the NC group, *Firmicutes, Bacteroidetes* and *Proteobacteri*a were the dominant species, accounting for 19.64%, 20.77% and 50.29% of the total flora, respectively. The research shows that the two dominant microflora animal intestines are *Firmicutes* and *Bacteroidetes*^23^. Therefore, in the analysis of microbial community, the sum of these two kinds of bacteria accounts for more than 1/3 of the total microflora.

In the control group, *Proteobacteria* is the dominant flora, accounting for 50.3% of the total flora. *Proteobacteria* is the largest of the bacteria, which has important metabolic functions in anaerobic digestion^24^. It can use small molecular compounds such as glucose and butyrate to promote the degradation of amino acids and long-chain fatty acids. The main function of *Bacteroidetes* is to decompose carbohydrates and plant wall cell polysaccharides, while Thicklyophila can degrade organic substances such as starch and protein^25^. After different pH treatments, compared with the NC group, Firmicutes became the most dominant species. The reason is that Firmicutes can effectively resist the extreme environment, so it can adapt to the pH changes after acid/alkali treatment and enrich, lead to the population abundance increased after acid and alkali treatment. The abundance of Proteobacteria decreased from 50.3 to 10.29–22.55%, and it is no longer the dominant species in the bacterial community. Because Proteobacteria mainly plays the role of inducing cell lysis and releasing intracellular substances, this proves that the alkali-regulated AF process can reduce the organic P and degrade the insoluble protein^26^. Furthermore, it is reported that Spirochaetes is the main strain that promotes glucose fermentation to produce acetate, ethanol, H_2_, CO_2_ and other intermediates^27^.

In summary, the existence of microorganisms relies on various substrates as nutrients, decomposing and producing various substances that can be directly utilized by phosphorus accumulating microorganisms, such as short chain volatile fatty acids, as energy sources for anaerobic phosphorus release. At the same time, microorganisms can induce cell lysis and release intracellular substances, which in turn decompose into phosphorus elements, causing the phosphorus concentration in the supernatant to gradually increase. In addition, OP in sludge can be released into the liquid phase through sludge disintegration, and then converted into PO_4_^3–^ P through biological action. It can also be directly converted from solid phase OP to PO_4_^3−^–P through biomineralization.

### Effect of anaerobic fermentation on metal/heavy metal release

**Figure 5:**
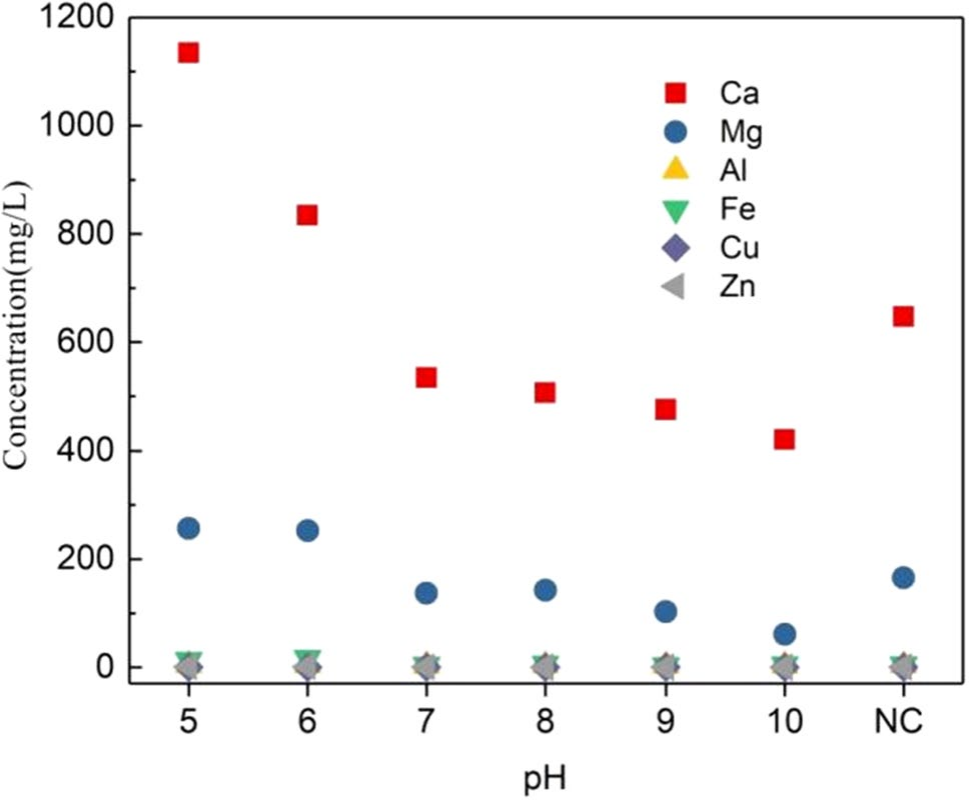
Effect of various metal components release under anaerobic fermentation conditions with different pH.

With the adjustment of pH, metal ions will inevitably be produced in the liquid phase. Through further analysis of metal ions, the mechanism of pH affecting the release of P in pig manure will be further clarified. As shown in the Fig. 5, under the condition of pH = 5, the contents of metals and heavy metals in the liquid phase are 1134.1 mg/L, 256.9 mg/L, 3.9 mg/L, 13.2 mg/L, 0.6 mg/L and 1.2 mg/L respectively. The content of major metals Ca^2+^, Mg^2+^ is the highest, and the content gradually decreases with the increase of pH. Under acidic conditions, Ca^2+^ mainly comes from the dissolution of AP, while Mg^2+^and Fe^3+^/Fe^2+^come from the dissolution of NAIP, which is consistent with the P composition in the solid phase. The dissolution of AP and NAIP causes the increase of the content of Mg^2+^ and Ca^2+^. With the increase of pH, the content of the two metals gradually decreased, because the combination of OH^−^ and Mg^2+^ subsequently formed metal hydroxide. The decrease of Ca^2+^ concentration may be due to the formation of apatite from Ca^2+^ and PO_4_^3−^ under alkaline conditions, indicating that alkaline conditions are not conducive to AP release, which is consistent with the results of solid-phase phosphorus fractionation extraction. In addition, the contents of Ca^2+^ and Mg^2+^ under alkaline conditions are lower than those of NC 646.8 mg/L and 165.3 mg/L, which also indicates that metal hydroxide is formed under alkaline conditions. The contents of heavy metals Cu^2+^ and Zn^2+^ in the liquid phase are very small, and they are 0.6 mg/L and 1.2 mg/L respectively at pH = 5. And its content decreased significantly with the increase of pH, and decreased to 0.1 mg/L and 0.3 mg/L respectively at pH = 10. Similar to the release law of major metals, heavy metals are released by acid leaching, and retained in the solid phase by forming hydroxide precipitation under alkaline conditions.

### The result evaluation of P recovery in two-chamber electrolytic cell

**Figure 6:**
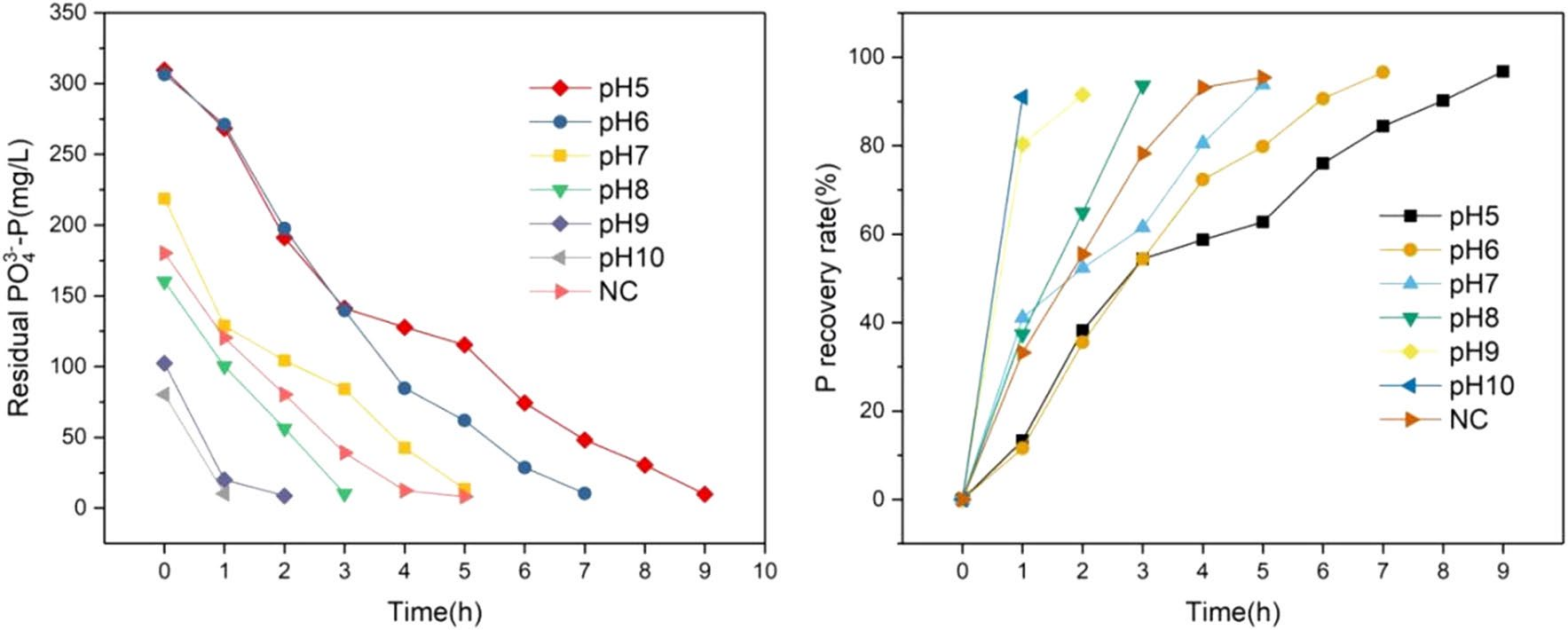
Phosphorus release characteristics under different pH conditions.

The supernatant after fermentation is analyzed and evaluated for P recovery by two-chamber electrolysis. The sampling interval is 1 h.The content of PO_4_^3−^- in the solution was determined after the samples passed through 0.45 μm membrane, and the P change trend was shown in the Fig. 6. The experimental results show that under different pH conditions, the residual P content in the solution is lower than 10 mg/L, and the P recovery rate is more than 90%, which proves that the effect of P recovery in the two-chamber electrolytic cell is excellent. P removal time is different under different pH conditions. The higher the P content, the longer the removal time, the higher the pH, and the shorter the removal time. However, P removal does not follow a linear change, indicating that P removal is not constant per unit time, which indicates that P removal is a dynamic imbalance process. In addition, under high pH conditions, the removal rate of P per unit time is faster. At the same time, it is observed in the reaction that the precipitate formed in the reactor is dark green, and rapidly turns to reddish brown when placed in the air. It is speculated that the product contains a large amount of Fe(OH)_2_, and the removal of P is mainly the adsorption of hydrogen oxides^28^. Previous studies have shown that when the pH value is fixed in the range of 6–7, the phosphate removal efficiency is the highest, and a large amount of precipitates into vivianite^29^.

### Change of metal composition after electrolytic supernatant

**Figure 7:**
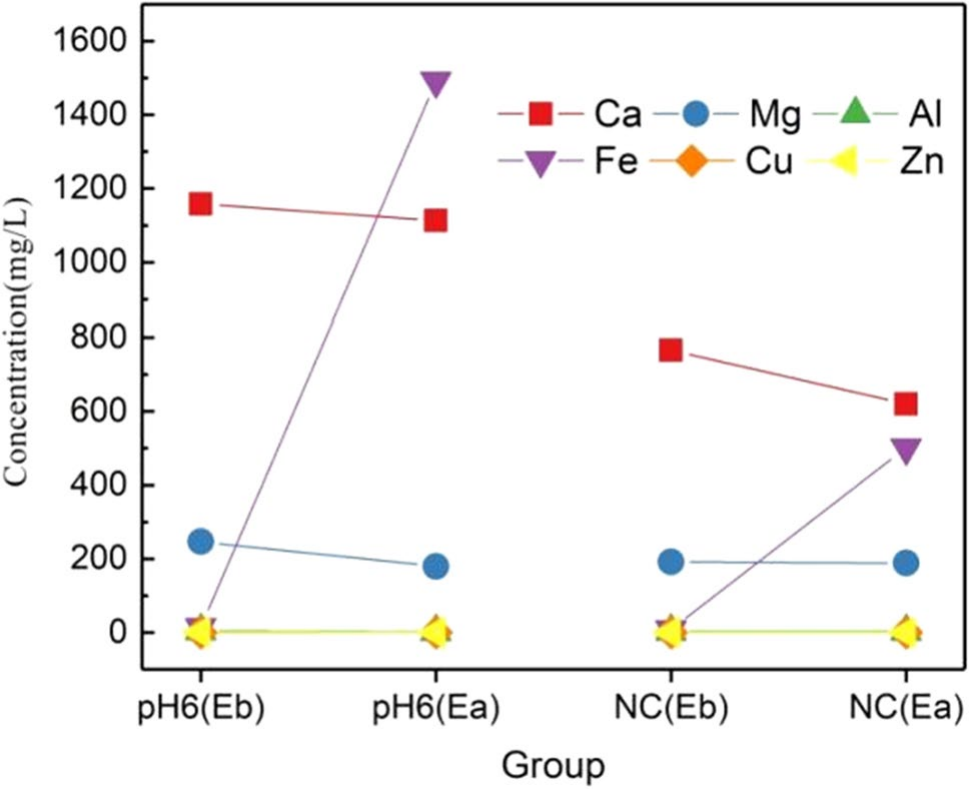
Change trend of metal content in liquid phase before and after electrolysis (Eb represents before electrolysis, Ea represents after electrolysis).

In order to explore the influence of the two-chamber electrolytic cell on the change of metal ions in the solution, the composition of metal ions in the electrolytic solution was analyzed. Previous studies have found that, the pH of the vivianite synthesized in the two-chamber electrolytic cell is stable at 6.29 ± 0.2. In this experiment, the final pH was maintained at 6.45 ± 0.1 under the condition of electrolysis of pH = 6. From the perspective of resource and economy, only the electrolytic results under this condition were quantitatively analyzed, and the supernatant after anaerobic fermentation of pH = 6 and NC was selected as the research object. After the completion of electrolysis, the residual P is 10.5 mg/L and 8.2 mg/L, and the P recovery rate is 96.58% and 95.43% respectively, which is also the maximum P recovery value in this experiment. The change of metal after electrolysis is shown in the Fig. 7: the result shows that under the condition of pH = 6, the content of Fe^3+^/Fe^2+^ increases from 13.98 to 1491.9 mg/L, and the content of Fe^3+^/Fe^2+^ in NC increases from 8.2 to 501.1 mg/L. The results show that the electrolytic iron anode of the two-chamber electrolytic cell has achieved the expected effect. Other metal components also decreased to varying degrees. Under pH = 6, Ca^2+^ and Mg^2+^ decreased by about 3.9% and 27% respectively. Under NC conditions, Ca^2+^ and Mg^2+^ decreased by 19% and 1.6% respectively. Al^3+^, Cu^2+^ and Zn^2+^ also decreased to varying degrees, but the change is small due to its low content.

The change of metal composition may be due to the formation of insoluble metal hydroxide attached to the surface of proton membrane due to the increase of local pH, which is considered as membrane pollution. However, this kind of precipitation is less than the detection threshold, so the existence of these metal ions is not detected in the EDS analysis of subsequent precipitation. Therefore, the removal of metal ions in the two-chamber electrolytic cell in this experiment is not obvious, and it is not suitable to remove certain metal ions by forming hydroxide precipitation, and it also proves that other metal ions do not participate in the formation of vivianite**.** During this reaction process, there may be the following reactions:

$${\text{Fe}} \to {\text{Fe}}^{2 + } + 2e^{ - }$$

$${\text{Fe}}^{2 + } \to {\text{Fe}}^{3 + } + e^{ - }$$

$$3{\text{Fe}}^{2 + } + 2{\text{PO}}_{4}^{3 - } + 8{\text{H}}_{2} {\text{O}} \to {\text{Fe}}_{3} \left( {{\text{PO}}_{4} } \right)_{2} \cdot 8{\text{H}}_{2} {\text{O}}$$

$${\text{Fe}}^{2 + } + 2{\text{OH}}^{ - } \to {\text{Fe}}\left( {{\text{OH}}} \right)_{2}$$

$${\text{Fe}}^{3 + } + 3{\text{OH}}^{ - } \to {\text{Fe}}({\text{OH}})_{3}$$

### Characterization of precipitation

#### XRD

**Figure 8:**
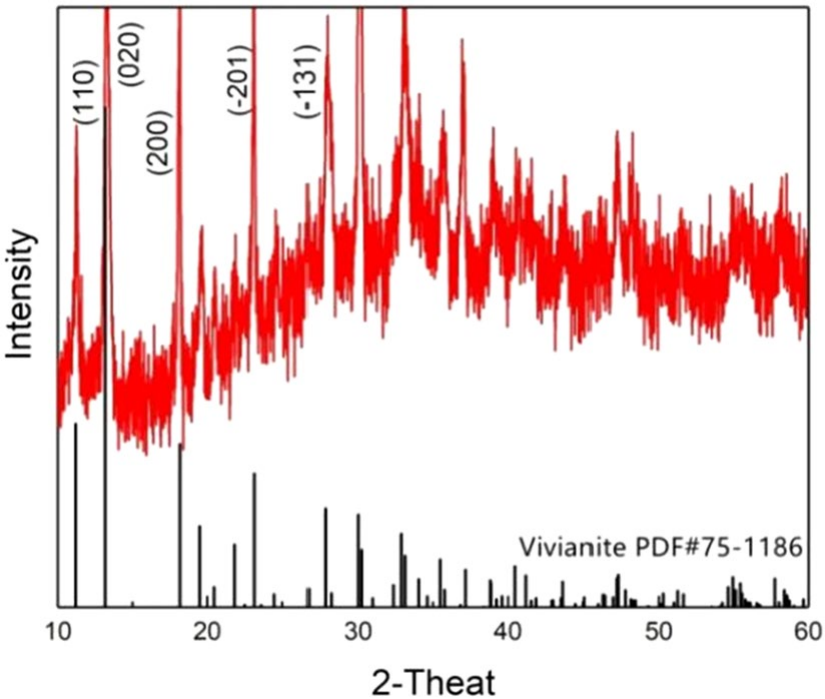
XRD spectrogram of sediment recovery from electrolytic supernatant.

XRD analysis was carried out for the crystals produced after electrolytic precipitation. The results are shown in the Fig. 8. It is observed that the crystal has obvious characteristic peak at 11.05°, 13.05°, 18.05°, 23.14°, 27.85°, 30.06°, 33.02°, which is completely consistent with the standard.

vivianite XRD card (PDF#75–1186), proving that the crystal is vivianite.

#### FTIR (Fourier transform ioncyclotron resonance)

**Figure 9:**
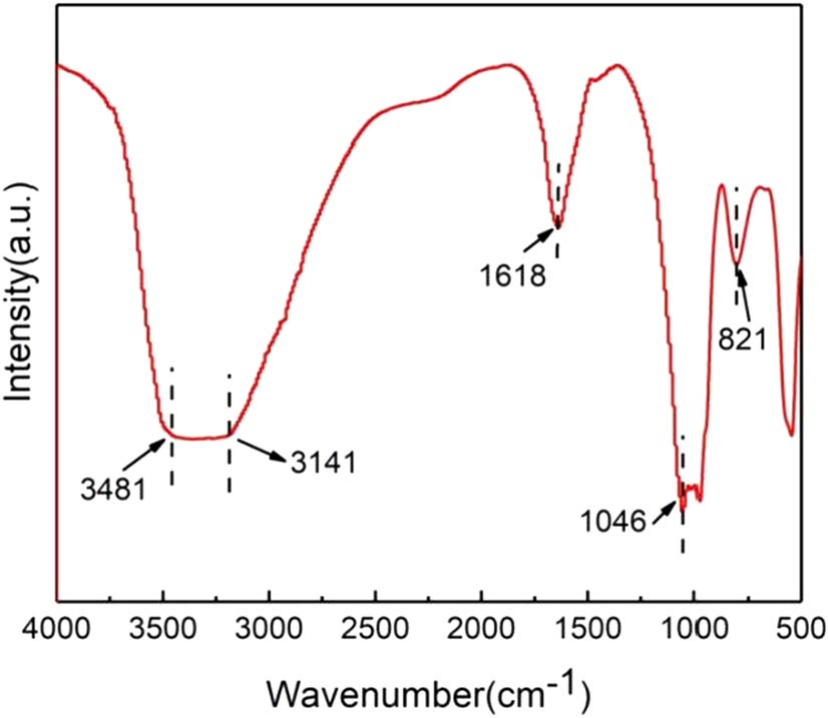
FTIR spectrogram of precipitate recovered from electrolytic supernatant.

The functional groups of the precipitate were further analyzed by infrared spectroscopy. The results are shown in the Fig. 9: the peak in the (3800–2600 cm^−1^) band including 3480 cm^−1^ was interpreted as the vibration of OH^−^ in Fe(OH)_3_. The existence of this band confirmed the partial oxidation of vivianite in the sediment. In the (1700–1500 cm^−1^) absorption band range, the maximum absorption band is 1618 cm^−1^, which corresponds to the deformation vibration of H_2_O molecule, and confirms the existence of water in the molecular structure. There are two very strong absorption bands near 1046 and 974 cm^−1^ correspond to the triple degenerate valence vibration F_2_(2)(V_3_) of distorted tetrahedron [PO4]^3−^. The weaker band near 940 cm^−1^ is related to the fully symmetric valence vibration A_1_(V_1_) of tetrahedron [PO4]^3−^^30^.The strong band at 821 cm^−1^ corresponds to the translational motion of H_2_O molecule. The existence of functional groups confirms that the main phase of the material is vivianite.

#### SEM–EDS

**Figure 10:**
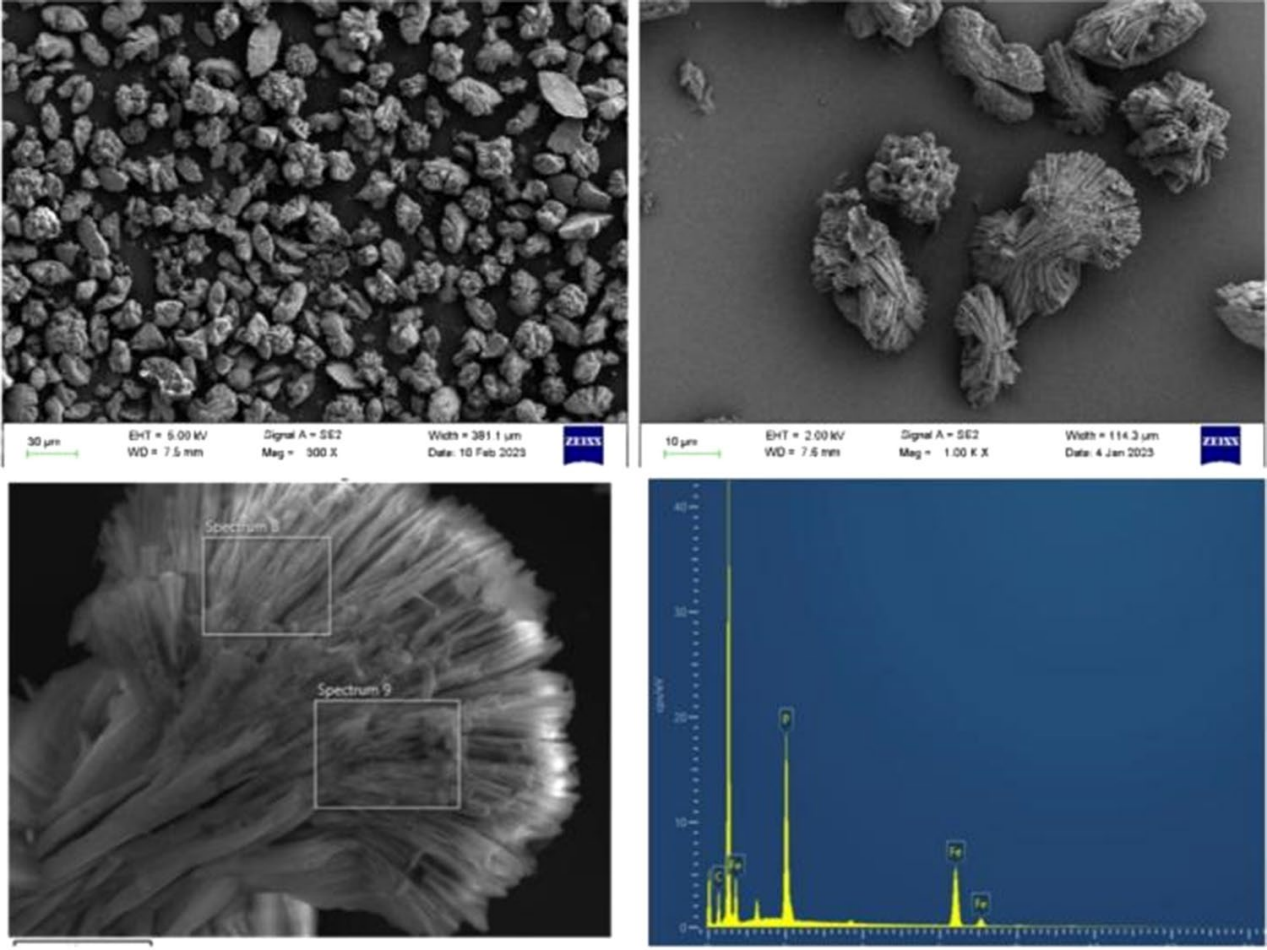
SEM–EDS diagram of the precipitation products of the supernatant with pH of 6.0.

The morphology characterization and element analysis of the precipitates are shown in the Fig. 10: in the SEM morphology analysis, it is observed that most of the precipitates are stacked into agglomerates in the form of branched and plate-like, which are closely related to the reaction conditions^31,32^. According to the importance of elements O, Fe and P of EDS (Figure S2), the molar ratio of Fe: P calculated by element content is 1.27 (Table S1), which is slightly less than the theoretical molar ratio of 1.5. This can be attributed to the adsorption of iron oxide on phosphate^33^. According to formula (2), 84.66% of the recovered product is vivianite.

## Conclusion

The anaerobic fermentation experiment of pig manure showed that pH regulation had obvious effect on anaerobic fermentation of pig manure, and the effect of promoting fermentation followed acid > NC > alkaline. Therefore, for the subsequent anaerobic fermentation, under the condition of avoiding acid and alkali pollution, neutral fermentation can be used, and under this pH condition, the phosphate recovery rate is the best, which is also the theoretical pH range of vivianite synthesis. For the contribution of different P components, acidic conditions are more suitable for the release of NAIP and AP, and the NC group is also higher than the P contribution under alkaline conditions. The content of Ca^2+^ and Mg^2+^ ions gradually decreases with the increase of pH, and the content of Ca^2+^ and Mg^2+^ ions is high under acidic conditions due to the acid dissolution of metal ions, while the decrease of ion content under alkaline conditions is due to the formation of metal hydroxide precipitation. The experiment of electrolytic recovery of the supernatant after fermentation shows that the two-chamber electrolytic cell has excellent potential in P recovery, and the P recovery rate is above 90%. XRD, FTIR, SEM–EDS analysis and qualitative and quantitative analysis of the recovered product confirmed that the product was mainly vivianite. 84.66% of vivianite was recovered.

### Supplementary Information

Supplementary Information.

## Data Availability

The datasets used and/or analyzed during the current study are available from the corresponding author on reasonable request.
