# Supplementary material for: Study on anaerobic phosphorus release from pig manure and phosphorus recovery by vivianite method
Source: Sci Rep. 2023 Sep 26;13:16095. doi: 10.1038/s41598-023-43216-5 (PMC10522647; doi:10.1038/s41598-023-43216-5)
Supplement: Supplementary file 1 — Supplementary Information. [file 41598_2023_43216_MOESM1_ESM.docx]

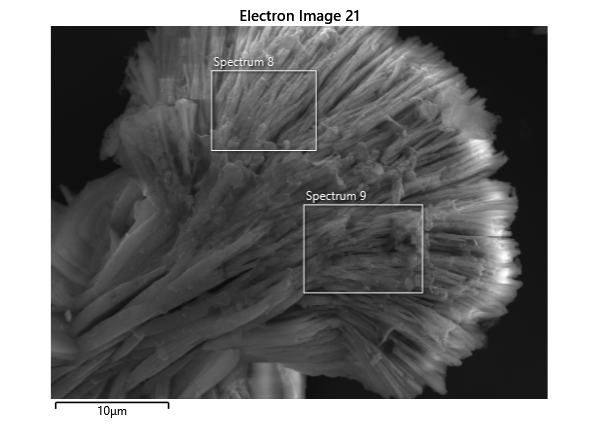


Figure S1: EDS diagram of sediment surface structure


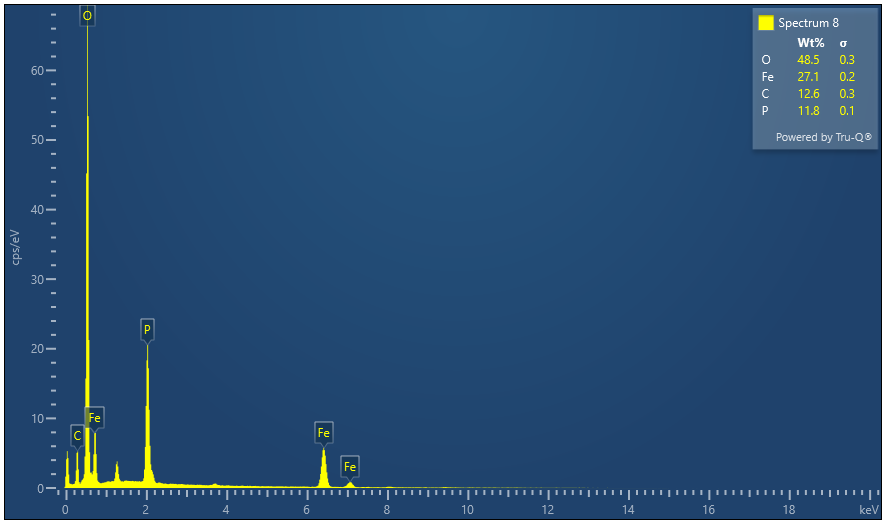


Figure S2: Surface energy dispersion spectrum of sediment

| **Spectrum 8** | | | | | |
| --- | --- | --- | --- | --- | --- |
| Element | Apparent Concentration | k Ratio | Wt% | Wt% Sigma | Factory Standard |
| C | 6.49 | 0.06493 | 12.56 | 0.28 | Yes |
| O | 159.90 | 0.53807 | 48.51 | 0.25 | Yes |
| P | 36.12 | 0.20204 | 11.78 | 0.11 | Yes |
| Fe | 49.02 | 0.49015 | 27.15 | 0.24 | Yes |
| Total: |  |  | 100.00 |  |  |

Table S1: EDS element content data under the surface of sediment at pH=6.0

**SMT method:**

Phosphorus fractionation extraction: Solid-phase phosphorus component analysis was performed using the SMT method. The steps are as follows:

(1) Total phosphorus (TP): Weigh 0.2 g of the freeze-dried sample in a porcelain crucible and place it in a muff furnace at 450°C for 3 h. After cooling, transfer the burned product to a centrifuge tube with 20 mL of 3.5 M HCl solution, fix it in a constant temperature shaker at 25°C, and shake for 16 h. After completion, centrifuge at 4000 r for 15 min and measure the PO_4_^3—^P concentration in the liquid phase.

(2)Inorganic phosphorus (IP): Weigh 0.2 g of the freeze-dried sample in a centrifuge tube, add 20 mL of 1.0 M HCl solution, and follow the remaining steps of TP.

(3) Organic phosphorus (OP): Wash the residue after IP determination with 12 mL of deionized water, shake for 5 min, centrifuge at 4000 r for 15 min, discard the wash liquid, and transfer the washed residue into a 20 mL porcelain crucible. After drying in a water bath, burn it in a muff furnace at 450°C for 3 h. After cooling, transfer the sample to a centrifuge tube with 20 mL of 1.0 M HCl solution, and follow the remaining steps of TP.

(4) Non-apatite inorganic phosphorus (NAIP): Weigh 0.2 g of the freeze-dried sample in a centrifuge tube, add 20 mL of 1.0 M NaOH solution, shake at a constant temperature for 16 h, centrifuge at 4000 r for 15 min, take 10 mL of the supernatant and add 4 mL of 3.5 M HCl solution, mix vigorously for 20 s, let it stand for 16 h, and measure the PO_4_^3-^-P concentration in the liquid phase.

(5) Apatite inorganic phosphorus (AP): Wash the residue after NaOH extraction with saturated NaCl solution three times, add 20 mL of 1.0 M HCl solution, and follow the remaining steps of TP.
